# Preparing Kaempferol Nanosuspension (KNS) using High Pressure Homogenization (HPH) technique

**DOI:** 10.1186/1753-6561-9-S7-A22

**Published:** 2015-10-27

**Authors:** Yew Sheng Qian, Ravindran Hari Kumar, Venkata Srikanth Meka, Senthil Rajan Dharmalingam

**Affiliations:** 1 International Medical University, Kuala Lumpur, Malaysia; 2 University Putra, Selangor, Malaysia

## Background

During the last decades, researchers have provided a plethora of therapeutic uses of kaempferol, including anti-cancer, anti-inflammatory, neuroprotective, anti-oxidative and anti-oestrogenic activities, making kaempferol a valuable compound [1]. However, being a poor water-soluble compound, kaempferol often has insufficient solubility and bioavailability [2].To find a solution for this limitation, this research project mainly focuses on formulating a kaempferol nanosuspension (KNS) using High Pressure Homogenisation (HPH), followed by performing physiological characterization. Kaempferol nanosuspensions are supposed to have a better bioavailability in animal models or even human subjects in future experiments, thereby reducing oral drug dosage required by consumers.

## Methods

A weighed quantity of pure kaempferol (1%w/v) was dispersed in ultrapure water. The mixture had undergone magnetic stirring at 3000rpm for 30 mins. Then the mixture was sonicated using an ultrasonic probe sonicator. The amplitude was set at 100% for 1 minute. Finally, the mixture was homogenised by a high pressure homogeniser at 500 Bar, 1100 Bar and 1700 Bar for 10, 20 and 20 cycles respectively. The particle size, chemical and physical characteristics of kaempferol nanosuspension being produced was compared with that of pure kampferol. The characterization techniques include Differential Scanning Calorimetry, X-ray Diffraction, Transmission Electron Microscopy and Fourier Transform Infrared Spectroscopy. Data was expressed as mean ±S.E.M. Significance levels for comparison.

## Results

The KNS produced by HPH undergone a significant reduction in particle size (from 2µm to 400nm), but at the same time, maintaining its original chemical and physical characteristics, suggesting that this nanosized kaempferol has similar therapeutic effects as the pure drug.

## Conclusions

Nanosuspensions produced by HPH represent an optimal solution for many poorly soluble substances and, therefore, they are still considered as a formulation of first choice due to the simplicity of the system.

## References

[B1] Calderon-Montano JM Burgos-Moron E Perez-Guerrero C Lopez-Lazaro M A review on the dietary flavonoid kaempferol Mini Rev Med Chem 2011 11 4 298 344 10.2174/138955711795305335 21428901

[B2] Muller RH Benita S Emulsion and nanosuspension for the formulation of poorly soluble drugs Medpharm Scientific, Stuttgart 1998 143 143

